# A randomized controlled trial of vitamin D dosing strategies after acute hip fracture: No advantage of loading doses over daily supplementation

**DOI:** 10.1186/1471-2474-12-135

**Published:** 2011-06-20

**Authors:** Alexandra Papaioannou, Courtney C Kennedy, Lora Giangregorio, George Ioannidis, Janet Pritchard, David A Hanley, Leonardo Farrauto, Justin DeBeer, Jonathan D Adachi

**Affiliations:** 1 McMaster University, HHSC, St. Peter's Hospital Juravinski Research Centre, 88 Maplewood Avenue Hamilton, ON L8M 1W9, Canada; 2 University of Waterloo, Department of Kinesiology, University of Waterloo, 200 University Ave W., Waterloo ON N2L 3G1, Canada; 3 McMaster University, 501-25 Charlton Ave E, Hamilton ON L8N 1Y2, Canada; 4 McMaster University, HHSC, St. Peter's Hospital Juravinski Research Centre, 88 Maplewood Avenue Hamilton, ON L8M 1W9, Canada; 5 University of Calgary, University of Calgary Health Sciences Centre, 3330 Hospital Drive N.W., Calgary AB T2N 4N1, Canada; 6 McMaster University, Henderson Hospital, 711 Concession Street, Section M, First Floor, Room 37, Hamilton ON L8V 1C3, Canada; 7 McMaster University, 1 Young St. Suite 307, Hamilton ON L8N 1T8, Canada; 8 McMaster University, 501-25 Charlton Ave E, Hamilton ON L8N 1Y2, Canada

## Abstract

**Background:**

There remains uncertainty regarding the appropriate therapeutic management of hip fracture patients. The primary aim of our study was to examine whether large loading doses in addition to daily vitamin D offered any advantage over a simple daily low-dose vitamin D regimen for increasing vitamin D levels.

**Methods:**

In this randomized controlled study, patients over age 50 with an acute fragility hip fracture were enrolled from two hospital sites in Ontario, Canada. Participants were randomized to one of three loading dose groups: placebo; 50,000 IU vitamin D_2_; or 100,000 IU D_2_. Following a placebo/loading dose, all patients received a daily tablet of 1,000 IU vitamin D_3 _for 90 days. Serum 25-hydroxy vitamin D (25-OHD) was measured at baseline, discharge from acute care (approximately 4-weeks), and 3-months.

**Results:**

Sixty-five patients were enrolled in the study (44% male). An immediate rise in 25-OHD occurred in the 100,000 group, however there were no significant differences in 25-OHD between the placebo, 50,000 and 100,000 loading dose groups after 4-weeks (69.3, 84.5, 75.6 nmol/L, p = 0.15) and 3-months (86.7, 84.2, 73.3 nmol/L, p = 0.09), respectively. At the end of the study, approximately 75% of the placebo and 50,000 groups had reached the target therapeutic range (75 nmol/L), and 44% of the 100,000 group.

**Conclusions:**

In correcting vitamin D insufficiency/deficiency in elderly patients with hip fracture, our findings suggest that starting with a lower daily dose of Vitamin D_3 _achieved similar results as providing an additional large loading dose of Vitamin D_2_. At the end of the study, all three groups were equally effective in attaining improvement in 25-OHD levels. Given that a daily dose of 1,000 IU vitamin D_3 _(with or without a loading dose) resulted in at least 25% of patients having suboptimal vitamin D status, patients with acute hip fracture may benefit from a higher daily dose of vitamin D.

**Trial registration:**

Clinical Trials # NCT00424619

## Background

Vitamin D deficiency is a major risk factor for accelerated bone loss and fracture [1]. Insufficient vitamin D levels lead to reduced calcium absorption (particularly at low-to-moderate calcium intakes), elevated serum parathyroid hormone, and increased rates of bone resorption, which over time may lead to bone fracture [2]. The optimal level of serum 25-hydroxyvitamin D_3 _(25-OHD) for bone health has been defined by a consensus panel as 75 nmol/L [3]. This level has also been endorsed by Osteoporosis Canada [4] and the International Osteoporosis Foundation [5].

There is evidence from a number of meta-analyses that supplementation with at least 800 to 1,000 IU/day vitamin D_3 _reduces fractures [6,7] and falls [8]. The level of serum 25-OHD achieved is also an important consideration. It has been demonstrated that falls and fracture reductions are more consistently achieved when serum 25-OHD levels are at least 60 nmol/L and 75 nmol/L, respectively [9-11].

Patients with hip fracture are at high risk for a recurrent hip fracture or other fragility fractures [12] and should be targeted for osteoporosis treatment (i.e. bisphosphonate or other antiresorptive). Before hip fracture patients are initiated on bisphosphonate therapy, an important consideration is whether 25-OHD is at a therapeutic level [13,14]. The majority of patients with hip fracture have been found to have lower serum concentrations of 25-OHD than those without a fracture [15-19].

There remains uncertainly regarding the appropriate management of hip fracture patients in the acute fracture period, particularly for patients who are severely vitamin D deficient. Several studies have examined various vitamin D dosing strategies in patients with fractures and/or elderly cohorts [20-24]. However, the focus of previous studies was comparing the timing of dosing (i.e., daily versus weekly versus monthly). Whether supplementing patients with a large loading dose of vitamin D_2 _in addition to daily vitamin D_3 _offers any advantage over simply prescribing daily vitamin D_3 _is yet to be determined. Thus, the primary aim of our study was to compare change in 25-OHD levels after supplementation with daily vitamin D_3 _plus one of three loading dosing regimens: 1) placebo bolus 2) 50,000 IU vitamin D_2 _3) 100,000 IU vitamin D_2_.

## Methods

### Participants

Between October 2007 and April 2009, consenting and eligible patients over age 50 with an acute fragility hip fracture (defined as femoral neck, trochanteric, subtrochanteric or subcapital) which was the result of a minimal trauma accident, defined as a fall from standing height or less, were enrolled from two large academic hospital sites in Hamilton, Ontario, Canada. Patients were not eligible if they had pelvic fractures; pathological fractures secondary to malignancy or intrinsic bone disease (e.g. Paget's disease); pre-existing bone abnormality; cancer in the past 10 years likely to metastasize to bone; renal insufficiency (creatinine <30 mls/min); renal stones in past 10 years; hypercalcemia (primary hyperparathyroidism; granulomatous diseases); hypocalcemia; stroke within the last 3 months; or had taken hormone replacement therapy, calcitonin, bisphosphonates, raloxifen, or parathyroid hormone during the previous 24 months. Patients admitted from long-term care facilities/nursing homes were also excluded.

### Study Protocol

This was a parallel, double-blind, randomized controlled study. The central in-patient pharmacy at McMaster University Medical Centre coordinated the randomization procedure and the distribution of study drugs. Patients were randomized in blocks according to a computer-generated randomization list. The medication treatment group was concealed and all participants, study coordinators, physicians, staff, and caregivers were blinded to treatment group allocation. An overview of the study was provided to potential participants by surgical staff during routine examination (prior to or following hip fracture repair). Study coordinators followed-up with patient enrolment and informed consent within 7 days pre or post-operatively.

Participants were randomized to one of three groups which differed only by the oral bolus loading dose received on day 1: placebo bolus, 50,000 IU vitamin D_2_, or 100,000 IU vitamin D_2_. Following the one-time bolus dose at baseline, patients in all groups received a daily tablet of 1,000 IU vitamin D_3 _for 90 days. Vitamin D bolus doses and tablets were dispensed by nursing staff while in-hospital. Prior to discharge from hospital, study coordinators provided participants with the remainder of the 1,000 IU vitamin D_3 _tablets to be taken daily and reviewed medication instructions. Additional daily vitamin D_3 _(1,000 IU) pills were provided to participants if their 3-month examination date was extended. Adherence data for the inpatient period was provided by the hospital pharmacist, and pill counts for the post-discharge period were conducted at the 3-month clinic appointment by the study coordinators. Adherence was defined as taking at least 80% of dispensed daily vitamin D_3 _pills.

Vitamin D_3 _(cholecalciferol) is the molecule that is synthesized in the skin in response to ultraviolet B light exposure, while vitamin D_2 _(ergocalciferol) is derived from irradiation of certain fungi. Both vitamin D_2 _and vitamin D_3 _go through the stages of metabolism to create the active form of vitamin D (1,25-dihydroxyvitamin D), there is support that vitamin D_2 _may not be used as efficiently as vitamin D_3 _in human physiology [25-27].

### Study Measures

#### Laboratory

Serum 25-OHD was measured at baseline, discharge from acute care at approximately 4-weeks, and at a follow-up study visit at approximately 3-months. The measurements may have been several days from the actual 4-week (mean = 26.3, standard deviation = 7.9) and 3-month (mean = 99.0, standard deviation = 25.0) time points but for simplicity they will be referred to as "4-weeks" and "3 months" throughout the paper. Baseline blood samples were drawn in-hospital; additional venipunctures were performed prior to hospital discharge, and at 3-months either in-hospital (for patients remaining in acute care or rehabilitation) or at the out-patient clinic visit. In addition to 25-OHD, serum calcium, parathyroid hormone (PTH), phosphate, albumin, alkaline phosphatase, hemoglobin, and creatinine were assessed at baseline.

Serum 25-OHD was analyzed with the DiaSorin, 25-hydroxyvitamin D radioimmunoassay (Stillwater, Minnesota 55082-0285, U.S.A). The central laboratory analyzed all 25-OHD tests with the exception of three patients who had their final 25-OHD analyzed at alternate laboratories for logistical reasons.

Clinical and demographic characteristics were abstracted from the patient chart and obtained from a patient or family interview for the purpose of describing the population, and included living situation, fracture history, family fracture history, ambulation status and aids, number medications, co-morbidities and bisphosphonate use. The Charlson Index [28] was also calculated, based on 19 categories of co-morbidity (defined by ICD-9-CM diagnosis and procedure codes) which are weighted and tabulated into an overall co-morbidity score. A higher score indicates greater disease burden. Height and weight were abstracted from the patient's chart or measured at the 3-month clinic appointment. This research was conducted in compliance with the Helsinki declaration and ethics approval was received from the Hamilton Health Sciences/McMaster University Faculty of Health Sciences Research Ethics Board. The Data Safety Monitoring Committee reviewed all adverse events.

### Statistical Analysis

We reported the results in accordance with the Consolidated Standards of Reporting Trials (CONSORT) criteria [29]. Study data were entered and managed using an ACCESS database (Microsoft Access 2000). Analyses were performed with SPSS v.18 (SPSS Inc. Chicago, Illinois) software package and SAS/Stat 9.1 (SAS Institute Inc., Cary, North Carolina). All analyses were intention to treat and included all available data (unless specified in sub-group analyses). Descriptive data are presented as means, standard deviation (SD), 95% confidence intervals (95% CI) for continuous variables and as proportions and percents for categorical data. Any outliers above or below 3 standard deviations were checked against original laboratory reports or chart documents. Box plots were constructed to display the distributions of 25-OHD values.

Baseline data were compared according to treatment groups using one-way ANOVA; Post hoc pairwise comparisons were made using Bonferroni tests. Pearson's chi-square or Fisher's exact tests were used to examine categorical variables as appropriate.

ANCOVA analyses were used to compare differences between groups on 25-OHD and functional measures, adjusting for relevant variables. To account for variation in when 25-OHD measures were collected, we controlled for time to measure (i.e. days from bolus administration to a 25-OHD measure). Multivariable linear regression analysis was used to examine the percent change in 25-OHD  between baseline and follow-up measures, adjusting for time to measure and baseline 25-OHD scores. The proportion of patients reaching the target 25-OHD therapeutic level (≥75 nmol/L) was also calculated and compared between groups using chi-square analysis. The criterion for statistical significance was set at alpha = 0.05.

## Results

**Figure 1:**
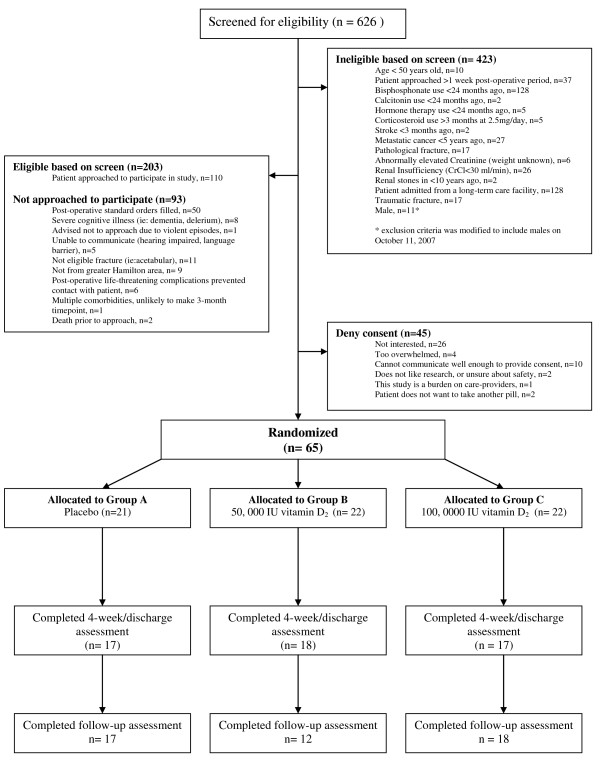
**Patients Screened**.

Of 626 patients screened for eligibility, 110 patients were approached to participate in the study as displayed in the CONSORT diagram (Figure 1). Of these, 45 denied consent and 65 were enrolled. Twenty-one of 65 (32%) patients were randomized to the placebo group, and 22 of 65 (34%) patients each to the 50,000 IU vitamin D_2_, and 100,000 IU vitamin D_2 _loading dose groups, respectively. One participant randomized to the placebo group was a screen failure (due to errantly receiving post-operative orders for vitamin D) and no data was collected on this patient.

Table 1 presents baseline clinical and laboratory characteristics. The mean age of the 100,000 group was significantly lower than the 50,000 group (p = 0.024). This is likely explained by the greater proportion of men in the 100,000 group as the men enrolled in our study tended to be younger than women (Table 1). Overall, 36 of 65 study participants (56%) were female. Fourteen of 65 participants (21%) had a previous atraumatic fracture after the age of 40 years documented, and 62% were ambulatory without aids prior to hip fracture.

**Table 1:** Characteristics of study sample at baseline, according to treatment allocation^†^

	Placebo n = 21	50,000 n = 22	100,000 n = 22	Missing Cases (Placebo/50,000/100,000)
Age in years	78.5 (10.3)	82.9 (8.7) *	73.9(12.4) *	1/0/0
Male (SD)	77.9 (8.4)	77.9 (11.2)	69.5 (12.9)	
Female (SD)	78.8 (11.5)	85.2 (6.4)	81.7 (6.6)	

Gender				1/0/0
Male (%)	7 (35)	7 (32)	14 (64)	
Female (%)	13 (65)	15 (68)	8 (36)	

Height, cm	163.8 (11.4)	162.9 (10.1)	169.7 (12.6)	2/3/2
Male (SD)	174.8 (10.2)	173.5 (4.7)	175.0 (10.3)	
Female (SD)	158.7 (7.9)	156.7 (6.3)	158.2 (7.3)	

Weight, kg	70.6 (25.4)	64.8 (18.1)	72.1 (15.8)	2/2/2
Male (SD)	89.1 (30.3)	79.4 (12.7)	76.1 (10.8)	
Female (SD)	62.1(18.4)	57.0 (15.8)	64.6 (21.4)	

Prior Smoking (%)	9 (50)	7 (39)	11 (58)	3/4/3

Previous fracture documented (%)	3 (15)	5 (23)	6 (27)	1/0/0

Prior vitamin D use (%)	4 (20)	1 (4.5)	4 (18)	1/0/0

Prior calcium use (%)	5 (25)	2 (9)	4 (18)	1/0/0

Number of medications at admission (SD)	6.2 (4.6)	6.0 (3.8)	5.0 (4.4)	1/0/0

Charlson index (SD)	1.30 (1.13)	0.82 (1.18)	0.55 (0.80)	2/0/0

Ambulatory without aids prior to admission (%)	8 (44)	13 (72)	13 (68)	3/4/3

Total calcium, mmol/L (SD)	2.10 (0.14)	2.12 (0.11)	2.08 (0.14)	1/1/0

Hb, g/L (SD)	108.6 (15.2)	102.2 (14.3)	107.9 (17.4)	1/0/0

Albumin, g/L (SD)	34.1 (6.0)	33.0 (5.8)	32.4 (5.4)	1/5/4

Creatinine, umol/L (SD)	73.7 (37.0)	70.5 (23.8)	77.4 (26.1)	1/0/0

Phosphate, mmol/L (SD)	0.94 (0.27)	0.86 (0.22)	0.97 (0.24)	1/0/0

Alkaline phosphatase, U/L (SD)	98.6 (37.8)	78.7 (26.2)	79.4 (51.1)	1/0/0

Parathyroid Hormone (PTH), pmol/L (SD)	4.20 (2.12)	5.35 (3.01)	5.10 (3.91)	1/0/0

*Significant difference between groups, p <0.05.

^†^Refers to loading dose of vitamin D_2 _at baseline; all groups received 1,000 IU vitamin D_3 _/daily following loading dose.

Values are the mean (SD), or number (%)

**Figure 2:**
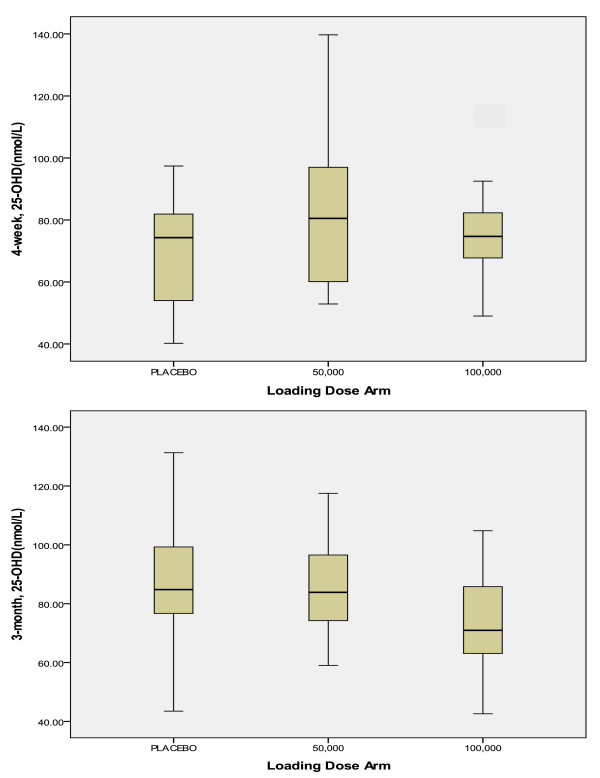
**Distribution of serum 25-OHD values by treatment group at Week 4 and 3 months**.

The box plots in Figure 2 illustrate the distribution of serum 25-OHD values by treatment group at 4-weeks and 3-months. There were no significant differences in mean serum 25-OHD between groups at 4-weeks or 3-months (Table 2). By 4-weeks, 47-59% of patients had reached the target therapeutic range (≥ 75 nmol/L). By 3-months, approximately 75% of the placebo and 50,000 groups had reached therapeutic range, and only 44% of the 100,000 group, but the difference between groups in the proportion of patients in the therapeutic range was not statistically significant (Table 2).

**Table 2:** Mean (95% CI) 25-OHD and percent reaching 75 nmol/L (N = 65)

	Placebo	50,000	100,000	p-value
**Baseline (n = 59)**				
Mean 25-OHD, unadjusted				
serum collection at -3 to 0 days, n = 34	58.7 (43.4-74.0) n = 7	52.3 (37.4-67.2) n = 14	44.3 (30.8-57.8) n = 13	0.39
serum collection at days 1-3, n = 25	41.6 (29.3-53.9) n = 11	59.0 (28.4-89.6) n = 6	76.0 (49.1-102.8) n = 8	0.03^†^

Mean 25-OHD, adjusted for time to measure, n = 59^‡ ^	46.7 (34.8-58.6) n = 18)	53.5 (42.3-64.8) n = 20	58.4 (47.3-69.5) n = 21	0.37

**4-week (n = 50)**				
Mean 25-OHD, unadjusted	69.8 (59.8-79.8) n = 17	84.1 (69.3-98.9) n = 17	75.6 (67.6-83.6) n = 16	0.17
Mean 25-OHD, adjusted for time to measure*	69.3 (58.4-80.2) n = 17	84.5 (73.7-95.3) n = 17	75.6 (64.5-86.8) n = 16	0.15
Proportion ≥ 75 nmol/L	47.1%	58.8%	50.0%	0.76

**3-month (n = 47)**				
Unadjusted	85.0 (73.6-93.3) n = 17	85.2 (74.5-95.9) n = 12	74.3 (66.1-82.5) n = 18	0.17
Mean 25-OHD, adjusted for time to measure**	86.7 (77.6-95.9) n = 17	84.2 (73.6-94.9) n = 12	73.3 (64.5-82.1) n = 18	0.09
Proportion ≥ 75 nmol/L	76.5%	75%	44.4%	0.09

Baseline data (n = 59) was missing for two participants, and four outliers with 25-OHD taken at 6, 10 or 12 days were not included. 4-week data (n = 50) was missing for 13 participants, and two outliers with 25-OHD taken at <13 days were not included. 3-month data (n = 47) was missing for 18 participants. ^†^Significant difference between 100,000 and placebo (post-hoc). ^‡^Mean time between bolus vitamin D dose and baseline 25-OHD measure was 0.36 days. *Mean time between bolus vitamin D dose and 25-OHD at second measure was 28.1 days. **Mean time between bolus vitamin D dose and 25-OHD at third measure was 98.9 days.

In some cases, it was not possible to obtain a baseline serum sample until after the bolus dose. However, this limitation in timing allowed us to provide a descriptive comparison of the immediate effects of the dosing regimens. In this sub-group (n = 25) who had 25-OHD measured 1-3 days after administration of the bolus dose, there appeared to be an immediate rise in serum 25-OHD for patients taking the two loading doses, but the difference was only significant for the 100,000 vitamin D_2 _group compared with the placebo group (76.0 nmol/L versus 41.6 nmol/L, p = 0.03, Table 2).

In multiple linear regression analysis, controlling for time (between bolus dose and 25-OHD measures) and baseline 25-OHD, the percent change in 25-OHD was not significantly different between the treatment arms at 4-weeks or 3-months (data not shown). The mean percent change over 4-weeks was 43.3% (95% CI -0.29, 86.8), 100.3% (95% CI 59.3, 141.3), and 82.5% (95% CI 40.5, 124.5) for the placebo, 50,000 and 100,000 groups respectively. Over approximately 3-months, the mean percent change was 85.6% (95% CI 30.2, 141.0), 146.1% (95% CI 83.8, 208.4), and 68.1% (95% CI 17.1, 119.1).

### Safety

Five serious adverse events were reported: one in the placebo group, two in the 50,000 group, and two in the 100,000 group. The serious adverse events were: 2 deaths, 1 fractured hip, 1 pulmonary edema and myocardial infarction, 1 gangrenous left foot requiring amputation. All were judged unrelated to the study treatments by the Data Safety Monitoring Board.

### Adherence

Of the 64 patients who received study medication while in-hospital (until discharge to home/long-term care, study withdrawal, or death), 54 participants (83%) were adherent to daily 1,000 IU vitamin D_3 _(ie:, consumed at least 80% of tablets). The mean (SD) in-hospital compliance in the placebo, 50,000 IU and 100,000 IU vitamin D_2 _groups were 97.1 (7.6%), 86.8 (16.2%) and 89.5 (12.5%), respectively (p = 0.033). Of 47 participants who completed the final 25-OHD assessment, four participants (8%) completed the study in-hospital and are included in the above in-hospital adherence rate, and 13 (28%) did not bring their study pills to their clinic visit. Of the remaining participants who attended the final study visit, the mean (SD) at home compliance for those in the placebo, 50,000 IU and 100,000 IU vitamin D_2 _groups were 84 (14.5%), 96.1 (9.3%) and 91.8 (15.2%), respectively (p = 0.270).

## Discussion

Our findings reveal that daily vitamin D_3 _with or without a loading dose improves serum 25-OHD levels and that this result was seen as early as 4-weeks. Further, there were no significant differences in 25-OHD values between loading dose groups after 4-weeks or 3-months of daily 1,000 IU vitamin D_3 _therapy. Despite the significant increase in 25-OHD levels for all three groups, 1 in 4 participants in the placebo and 50,000 groups and 1 in 2 participants in the 100,000 group did not achieve the target therapeutic level (≥75 nmol/L) by 3-months. While not significantly different from the other two groups, the low number of participants in the 100,000 groups that did achieve the therapeutic level is an interesting finding and needs further investigation. Our findings suggest that daily 1,000 IU vitamin D_3 _may be nearly as or equally effective as providing an additional large loading dose of vitamin D_2 _for improving vitamin D levels in hip fracture patients, but many patients may still not achieve the therapeutic target. A daily dose of 2,000 IU vitamin D_3 _may be a better choice for this group of patients.

Daily vitamin D administration is at least as effective at elevating serum 25-OHD levels as compared with higher doses of vitamin D administered less frequently. In one study [22], after 8-weeks, no significant differences were found in serum 25-OHD levels for daily (1,500 IU vitamin D_3_), weekly (10,500 IU vitamin D_3_) or monthly (45,000 IU vitamin D_3_) dosing in 48 hip fracture patients. In addition, other investigators have demonstrated that a high does regimen of cholecalciferol 50, 000 IU daily for 10 days versus continuous low-dose cholecalciferol regimen of 3000 IU daily for 30 days, followed by 1000 IU daily for 60 days were both effective in increasing serum 25-OHD levels [30]. In a study of 40 elderly women, Pekkarinin et al. [20] found that daily 800 IU vitamin D_3 _was more effective in increasing serum 25-OHD levels than 97,333 IU vitamin D_3 _every four months (treatment groups were equivalent in terms of cumulative annual dose). In a cohort of elderly women with hip fractures, a group taking oral 800 IU vitamin D_3 _daily had similar 25-OHD after 12-months as a group taking a single injection of 300,000 IU of vitamin D_2 _(50 vs. 40 nmol/L) [31]. In a 4-month study of 338 elderly nursing home residents [23], daily 600 IU vitamin D_3 _was more effective than weekly 4200 IU vitamin D_3 _or monthly 18,000 IU vitamin D_3 _doses. While others investigators have found that daily regimens are at least as effective as monthly or weekly regimens assuming the same cumulative dose, our study is the first to show that providing a one time, large dose of vitamin D_2 _prior to daily vitamin D_3 _supplementation was not advantageous with respect to achieving optimal 25-OHD levels after several weeks.

In a sub-group of patients who were tested 1-3 days after receiving either the placebo or loading dose, an almost immediate rise in 25-OHD was observed in the loading dose groups compared with placebo, which was statistically significant for the 100,000 group. Other studies have seen similar initial increases in 25-OHD after large doses of vitamin D_3 _[45,000 IU [22]; 300,000 IU [32]] and vitamin D_2 _[300,000 IU [32]]. In a study with frail elderly patients, a loading dose of 500,000 IU vitamin D_3 _(without daily maintenance therapy) resulted in a rapid increase in 25-OHD in the first month, but progressively decreased reaching a mean of 59 nmol/L by 9-months [24]. In our study we found no significant differences in 25-OHD levels between groups after 4 weeks or 3 months after the placebo/vitamin D_2 _loading. It is possible that a large loading dose may not be as effective for long-term maintenance of optimal vitamin D levels because the large dose may induce increased 24-hydroxylase, causing an increased catabolism of both 25-OHD and 1,25-(OH)_2 _vitamin D. Therefore, less substrate is available for the production of 1,25-(OH)_2 _vitamin D, the biologically active form of vitamin D, and when produced it is more rapidly degraded. Our study suggests that a regimen of 1,000 IU vitamin D daily is as effective as vitamin D daily plus one loading dose, but further research is required to confirm whether the loading dose provides a benefit prior to the 4-week time point.

Our study demonstrated low 25-OHD levels in hip fracture patients. In addition, other investigators have found even lower serum levels, which highlights the need for vitamin D supplementations [33]. A key finding in the current study was that many patients had not achieved the therapeutic target of 75 nmol/L at the 3-month time-point. Previous studies comparing daily to weekly or monthly regimens have also reported sub-optimal vitamin D levels at the end of the study despite supplementation. In a cohort similar to ours, Ish-Shalom et al. [22] found that only half of patients with initial 25-OHD nmol/L below 50 nmol/L, and 80% of those with 25-OHD 50 nmol/L and above, achieved the therapeutic target (75 nmol/L) after 8-weeks of 1500 IU vitamin D_3 _daily. In nursing homes residents receiving 600 IU vitamin D_3 _daily, fewer than 40% achieved vitamin D levels of 75 nmol/L after 4 months [23]. Among elderly women with hip fractures receiving either 800 IU vitamin D_3 _daily or 97,333 IU monthly, 47% of the daily group and 28% of the monthly group achieved 75 nmol/L after 12 months [20]. Therefore other strategies such as a higher daily dose may need to be considered to enable a greater proportion of hip fracture patients to achieve optimal 25-OHD levels.

Achieving adequate 25-OHD levels after hip fracture is important from a therapeutic standpoint and may be needed to ensure optimal effectiveness of bisphosphonates [13,14]. In meta-analyses, a minimum daily dose of daily 700 IU vitamin D_3 _was considered necessary to prevent falls and osteoporotic fractures [34-36].

A number of limitations are acknowledged here. Although, we randomized patients into study groups, we found a significant difference in age among groups. Due to our sample size, and because some patients did not have a baseline measure prior to the bolus dose, we were unable to properly examine patients with greater baseline vitamin D deficiency or insufficiency. We did not examine the seasonal influence on 25-OHD levels since this was a short-term study and the majority of our patients were hospitalized for approximately a third or more of the study period. However, our results are consistent with other studies which have found that high dose vitamin D_3 _is not more effective than daily dosing [20,22,23]. D_3 _was not used as a loading dose because it is not available in Canada. Further research will need to be conducted to determine if a loading dose of D_3 _plus daily vitamin D_3 _is more effective as compared with a loading dose of D_2 _and to determine the most effective daily dose. In addition, parathyroid hormone levels were not measured so the relationship between the hormone and vitamin D levels could not be described. Examining the efficacy of vitamin D supplementation for the prevention of falls in the first 3-months post-hip fracture would be interesting given recent reports regarding the need for 25-OHD levels to be at 60 nmol/L for optimal fall prevention [37] and the recent observation that a single, once yearly, very large dose of vitamin D_3 _(500,000 IU) is associated with an increased risk of fall or fracture [38].

## Conclusions

Our findings reveal that a simple daily 1,000 IU vitamin D_3 _dosing regimen may be as effective as a regimen that adds a loading dose of vitamin D_2 _to daily vitamin D_3 _for increasing 25-OHD levels as early as 4-weeks. However, more than 25% of all study patients taking 1,000 IU vitamin D_3 _(with or without the loading dose) still did not achieve the target 25-OHD level of at least 75 nmol/L. Future studies should examine higher daily doses of vitamin D_3 _(i.e. 2,000 IU) as well as the benefits of an additional loading dose in patients who are severely deficient.

## Competing interests

**Alexandra Papaioannou: **Honoraria, grants received, or consultancies-Eli Lilly and Company, Merck Frosst, Amgen Inc, The Alliance for Better Bone Health (Procter & Gamble Pharmaceuticals and sanofi-aventis), Novartis Pharmaceuticals Corporation, Servier.

**Courtney C. Kennedy: **author declares she has no competing interests.

**Lora Giangregorio: **author declares she has no competing interests.

**George Ioannidis: **author declares he has no competing interests.

**Janet Pritchard: **author declares she has no competing interests.

**David A. Hanley: **Honoraria, grants received, or consultancies-Eli Lilly and Company, Merck Frosst, Amgen Inc, The Alliance for Better Bone Health (Procter & Gamble Pharmaceuticals and sanofi-aventis), Novartis Pharmaceuticals Corporation, Servier.

**Leonardo Farrauto: **author declares he has no competing interests.

**Justin DeBeer: **author declares he has no competing interests.

**Jonathan D. Adachi: **Honoraria, grants received, or consultancies: - Eli Lilly and Company, Merck Frosst, Amgen Inc, The Alliance for Better Bone Health (Procter & Gamble Pharmaceuticals and sanofi-aventis), Novartis Pharmaceuticals Corporation, GlaxoSmithKline Consumer Healthcare, Servier, Roche, Servier, Wyeth.

## Authors' contributions

AP: conceived the study, participated in the study design, and critically revised the manuscript for important intellectual content. CCK: participated in the study design, statistical analysis, interpretation of data, and drafted the manuscript. LG: participated in the study design and critically revised the manuscript for important intellectual content. GI: participated in the study design, statistical analysis, interpretation of data, and critically revised the manuscript for important intellectual content. JP: participated in the study design and critically revised the manuscript for important intellectual content. DAH: participated in the study design and critically revised the manuscript for important intellectual content. LF: participated in the study design and critically revised the manuscript for important intellectual content. JD: participated in the study design and critically revised the manuscript for important intellectual content. JDA: participated in the study design and critically revised the manuscript for important intellectual content. All authors read and approved the final manuscript.

## Pre-publication history

The pre-publication history for this paper can be accessed here:

http://www.biomedcentral.com/1471-2474/12/135/prepub
